# Utility of patient-derived lymphoblastoid cell lines as an *ex vivo* capecitabine sensitivity prediction model for breast cancer patients

**DOI:** 10.18632/oncotarget.9521

**Published:** 2016-05-20

**Authors:** Gladys Morrison, Divya Lenkala, Bonnie LaCroix, Dana Ziliak, Vandana Abramson, Phuong Khanh Morrow, Andres Forero, Catherine Van Poznak, Hope S Rugo, Rita Nanda, Peter H. O'Donnell, R. Stephanie Huang

**Affiliations:** ^1^ Section of Hematology/Oncology, The University of Chicago, Chicago, IL, USA; ^2^ Committee on Clinical Pharmacology and Pharmacogenomics, The University of Chicago, Chicago, IL, USA; ^3^ Vanderbilt University Medical Center and Vanderbilt-Ingram Cancer Center, Nashville, TN, USA; ^4^ Department of Breast Medical Oncology, The University of Texas, MD Anderson Cancer Center, Houston, TX, USA; ^5^ Department of Hematology and Oncology, University of Alabama at Birmingham, Birmingham, AL, USA; ^6^ Department of Medical Oncology, University of Michigan Comprehensive Cancer Center, Ann Arbor, MI, USA; ^7^ University of California San Francisco Helen Diller Family Comprehensive Cancer Center, San Francisco, CA, USA

**Keywords:** breast cancer, lymphoblastoid cell lines, patient-derived model, capecitabine, ex vivo model

## Abstract

Capecitabine is commonly used in treating breast cancer; however, therapeutic response varies among patients and there is no clinically validated model to predict individual outcomes. Here, we investigated whether drug sensitivity quantified in *ex vivo* patients' blood-derived cell lines can predict response to capecitabine *in vivo*. Lymphoblastoid cell lines (LCLs) were established from a cohort of metastatic breast cancer patients (*n* = 53) who were prospectively monitored during treatment with single agent capecitabine at 2000 mg/m^2^/day. LCLs were treated with increasing concentrations of 5′-DFUR, a major capecitabine metabolite, to assess patients' *ex vivo* sensitivity to this drug. Subsequently, *ex vivo* phenotype was compared to observed patient disease response and drug induced-toxicities. We acquired an independent cohort of breast cancer cell lines and LCLs derived from the same donors from ATCC, compared their sensitivity to 5′-DFUR. As seen in the patient population, we observed large inter-individual variability in response to 5′-DFUR treatment in patient-derived LCLs. Patients whose LCLs were more sensitive to 5′-DFUR had a significantly longer median progression free survival (9-month vs 6-month, log rank *p*-value = 0.017). In addition, this significant positive correlation for 5′-DFUR sensitivity was replicated in an independent cohort of 8 breast cancer cell lines and LCLs derived from the same donor. Our data suggests that at least a portion of the individual sensitivity to capecitabine is shared between germline tissue and tumor tissue. It also supports the utility of patient-derived LCLs as a predictive model for capecitabine treatment efficacy in breast cancer patients.

## INTRODUCTION

Capecitabine is an oral fluopyrimidine prodrug commonly used in treating breast and colorectal cancer patients. It is metabolized to 5′-deoxy-5-fluorouridine (5′-DFUR), which is further converted into the active metabolite 5-fluorouracil (5-FU) *in vivo* [1, 2]. In breast cancer patients, capecitabine is approved for the treatment of taxane-resistant metastatic breast cancer either as monotherapy or in combination with other chemotherapeutic agents. As monotherapy, and in combination with other chemotherapies, clinical trials have shown that capecitabine treatment results in a median overall survival range from 11–19 months and a median progression free survival (PFS) ranging from 3–9 months [3–6].

To maximize efficacy, studies have been conducted to identify patients who are likely to be non-responsive to capecitabine therapy prior to beginning a capecitabine-based regimen. Genetic variants that result in enzyme (e.g. thymidine phosphorylase (TP) and dihydropyrimidine dehydrogenase (DYPD)) activity deficiencies have also been shown to independently predict toxicity and sensitivity to capecitabine [7, 8]. More recently, the role of germline genetic variants in capecitabine sensitivity has been explored using a human cell-based model: the International HapMap lymphoblastoid cell lines (LCLs) [9].

Indeed, HapMap LCLs have previously been successfully used for pharmacogenomic discoveries of various drugs [10, 11]. Several genetic markers identified in this model have been validated *in vivo* in different cancer settings [9, 12]. However, HapMap LCLs were generated from apparently healthy donors with no known morbidities at the time of LCL establishment. Our study seeks direct answers to the question of whether establishing LCLs from diseased individuals is feasible and more importantly, how relevant/useful is a patient-derived LCL model in the clinical setting. In this study, we aimed at developing a blood-based *ex vivo* model for prediction of capecitabine sensitivity in breast cancer patients. Our rationale is that human peripheral blood is readily accessible and phenotypes obtained in this model can reflect both genetic and environmental effects on an individual. Specifically, we established LCLs from breast cancer patients and examined the relationship between phenotypes obtained in this *ex vivo* model and drug sensitivity phenotype (efficacy) obtained from the actual patients. Our hypothesis is that a patient-derived *ex vivo* LCL model can be used to predict a patient's clinical response to capecitabine. Furthermore, the establishment of LCLs from patients will provide materials for subsequent functional studies of gene and/or other genetic/epigenetic components without repeated clinical sampling, thus benefiting additional scientific discovery and validation.

## RESULTS

### Clinical response to capecitabine treatment

53 patients were included in this study and their ages ranged from 36 to 79 with a median age of 51 years old. The majority of patients were White (71%), with 17% African American and 1% Asian. Detailed clinical response/toxicity assessment results are shown in Table 1. Both short term and long term clinical responses were collected. The short term response was measured at 10–12 weeks capecitabine treatment; while the long term response was assessed using PFS, which range from 6 weeks to 32 months. Site of enrollment did not have an effect on response to capecitabine (data not shown).

**Table 1:** Patient characteristics and clinical response

Patient Demographics (*n* = 53)^*^		
Median Age (range)	51 (36–79) yrs	
Ethnicity	number	%
White	38	71%
Black	9	17%
Asian	2	1%
Other/undisclosed	5	5%
Clinical response^**^		
Short term response at 10–12 weeks		
CR	1 (2.9%)	
PR	7 (20%)	
SD	12 (34.3%)	
PD	10 (28.5%)	
ND	5 (14.3%)	

Number of patients with clinical response (percentages): Complete response (CR), Partial response (PR), Stable disease (SD), Progressive disease (PD), Not Disclosed (ND).

* 45 out of the 53 patients included in the correlative study had successful establishment of their LCLs.

** upon assessment of clinical data, some of these patients who had LCLs were lost to follow-up.

### Establishment of breast cancer patient-derived LCLs

Of the 53 patients who donated blood for our study, we successfully established LCLs for 45 individuals (success rate 85%). The reasons for failure to establish LCLs include improper method of blood storage/shipment (*n* = 4, blood received frozen and unable to isolate peripheral blood mononuclear cells (PBMCs)), shortage of EBV supplies from the vendor (*n* = 1), and failure to attain persistent proliferating status (*n* = 3). To assess technical variability and to verify that the variability of drug response observed among LCLs is due to inter-patient heterogeneity rather than possible heterogeneity caused by the establishment process, patient PBMCs were split into 2 vials and 2 independent LCLs from each individual donor were developed. The established LCLs were treated with increasing concentrations of 5′-DFUR (0–160 μM) and cell proliferation was assessed using Cell Titer Glo reagent. We found that in the majority of patient samples, there was no significant difference between the 2 batches of LCLs and their response to 5′-DFUR treatment (*p*-value > 0.05 from two-way ANOVA test between the drug sensitivity curves derived from each of the 2 batches of LCLs, Supplementary Figure 1). Thus, one LCL per patient was used for analysis. However, less than 8% of the patient derived LCLs (*n* = 4) showed variable 5′-DFUR sensitivity (defined by >15% variability) in the 2 independent LCLs created from the same individual. Both batches from each of these patients were removed from further analysis. Capecitabine response in patient-derived LCLs were also grouped based on their site of enrollment, and using one-way ANOVA, we found that there was no significant difference in their response to 5′-DFUR (*p* = 0.63).

### Patient-derived LCLs' sensitivity to 5′-DFUR

We observed that increasing concentrations of 5′-DFUR, correlated with a decrease in the cell viability of patient-derived LCLs (Supplementary Figure 2). Furthermore, large inter-individual variability e.g., 3-fold difference at 10 μM, was observed in these patient-derived LCLs within each treatment concentration. The median percent viability after 5′-DFUR exposure in the patient derived LCLs, ranged from 60 percent (10 μM) to 34 percent (160 μM).

### Relationship between drug sensitivity measured in patient-derived LCLs and that observed clinically

Clinical assessment and radiographic evaluation were performed on the majority of the patients at 12- week after initiation of capecitabine to assess their short term disease response. Response evaluation criteria in solid tumor (RECIST v1.1) criteria were applied and patient short term response was defined as complete response (CR), partial response (PR), stable disease (SD) or progressive disease (PD). We performed regression analysis between the 12-week RECIST response categorization and patients' 5′-DFUR sensitivity obtained in the LCL model (represented by Area under curve (AUC)). There is no significant association between patient RECIST-defined response at 12-week and response in their LCL model. There was, however, a trend showing patients with higher AUC in LCLs (representing resistance/less sensitivity to capecitabine) were more likely to have disease progression at 12-week (*p* = 0.086, Supplementary Figure 3).

**Figure 1:**
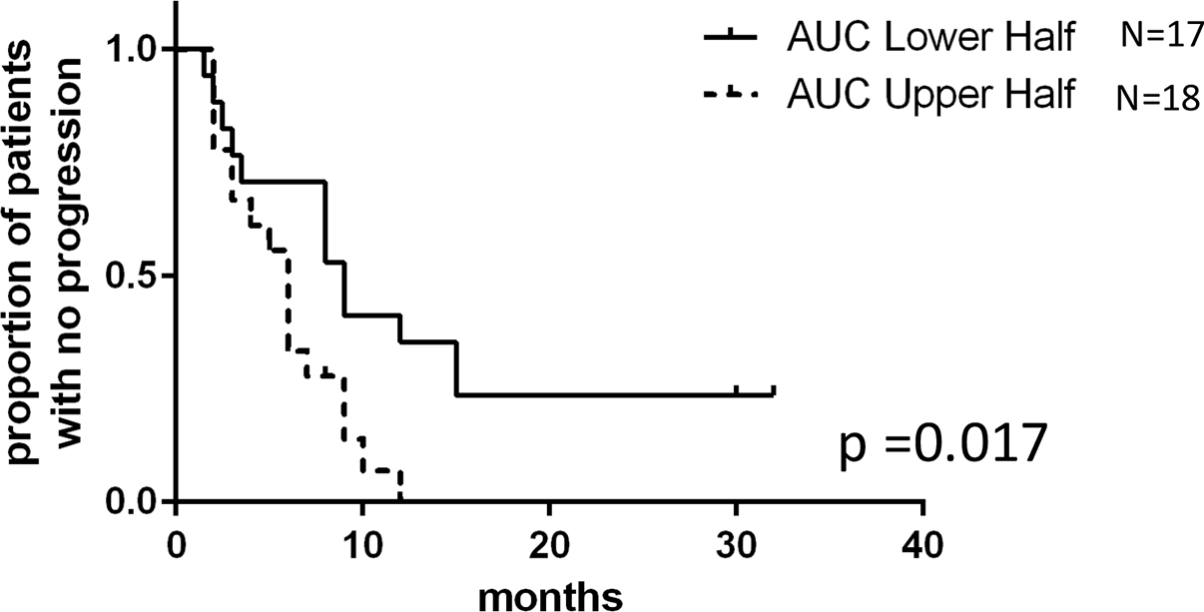
5′-DFUR sensitivity obtained from patient-derived LCLs can be used to predict breast cancer patients' PFS Kaplan-Meier curve represents PFS of stratified patients based on their LCL sensitivity to 5′-DFUR. 18 patients who have greater than mean *ex vivo* 5′-DFUR treatment AUC were compared to 17 patients who have lesser than mean *ex vivo* 5′-DFUR treatment AUC. Patients with lower AUC had a median 9 months PFS compared to those with higher AUC who only had a median of 6 months PFS (log rank *p* = 0.017).

We also evaluated the relationship between *ex vivo* phenotype and PFS (represented by time to tumor progression). Among the 45 patients from whom we successfully established LCLs, 10 patients were lost to follow-up. Using AUC obtained from the remaining 35 patient-derived LCLs, Cox regression and Kaplan-Meier analysis showed that patients with lower 5′-DFUR AUC (higher sensitivity) had significantly better outcomes (Figure 1). These patients whose LCLs were more sensitive to 5′-DFUR (defined as the lower halves of AUC distribution curve, Supplementary Figure 4), had a significantly longer PFS when compared to those patients whose LCLs were less sensitive to 5′-DFUR (median PFS: 9-month vs. 6-month, log rank *p* = 0.017). In addition, after adjusting for other known important prognostic clinical variables such as presence of hepatic metastases and age, the positive correlation between *ex vivo* phenotype and clinical survival remained significant (*p* = 0.025). Overall, these data suggest that *ex vivo* capecitabine sensitivity obtained from patient-derived LCL models may predict patients' clinical responses.

### *In vitro* assessment of capecitabine sensitivity between matching breast cancer cell lines and LCLs

**Figure 2:**
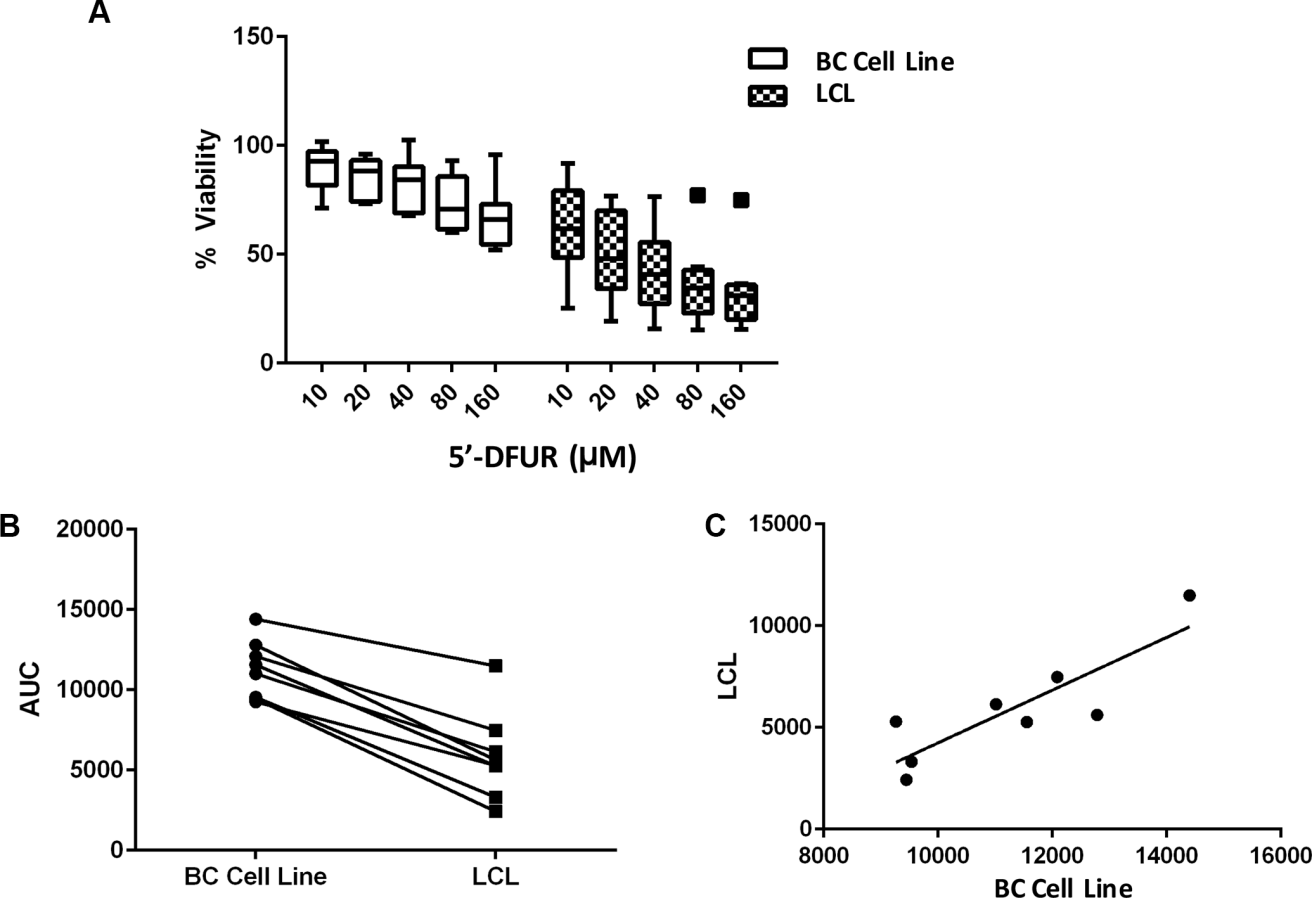
Cellular sensitivity to 5′-DFUR assessed in LCLs and their matched breast cancer cell lines (**A**) Decreased cellular viability was observed in both breast cancer (BC) cell lines and their matched LCLs when treated with increasing concentrations of 5′-DFUR for 72 hours. (**B**) Comparing cellular sensitivity to 5′-DFUR in both the BC cell lines and their matched LCLs. Student's *t*-test *p* = 0.0004 shows a significant difference in cellular response between BC cell lines and their matched LCLs in Area under the % viability curve (AUC) was calculated using trapezoidal rules. (**C**) AUC correlation between BC cell lines and their matched LCLs. Pearson correlation: *r* = 0.857 and *p* = 0.0067.

Given the observed correlation between LCL sensitivity to 5′-DFUR and patients' PFS on capecitabine treatment, we hypothesized that at least a portion of the individual sensitivity to capecitabine is shared between germline tissue and tumor. To test this hypothesis, we took advantage of a collection of previously-established matching LCLs (germline) and breast cancer cell lines (tumor) [13]. We performed 5′-DFUR sensitivity assays in both breast cancer cell lines and their matched LCLs derived from the same patient. Inter-individual differences in response to 5′-DFUR were observed in both LCLs and breast cancer cell lines (Figure 2A). Using a Student *T*-test to evaluate sensitivity between the 2 cell models, we found that LCLs in general were more sensitive to 5′-DFUR than breast cancer cell lines (Figure 2B, *p* = 0.0004). Interestingly, we found that 5′-DFUR sensitivity in LCLs was highly correlated with the sensitivity in their matched breast cancer cell lines (Figure 2C) with the higher the LCL sensitivity, the higher the tumor sensitivity to the same drug treatment (Pearson correlation coefficient *r* = 0.86 and *p* = 0.0067).

**Figure 3:**
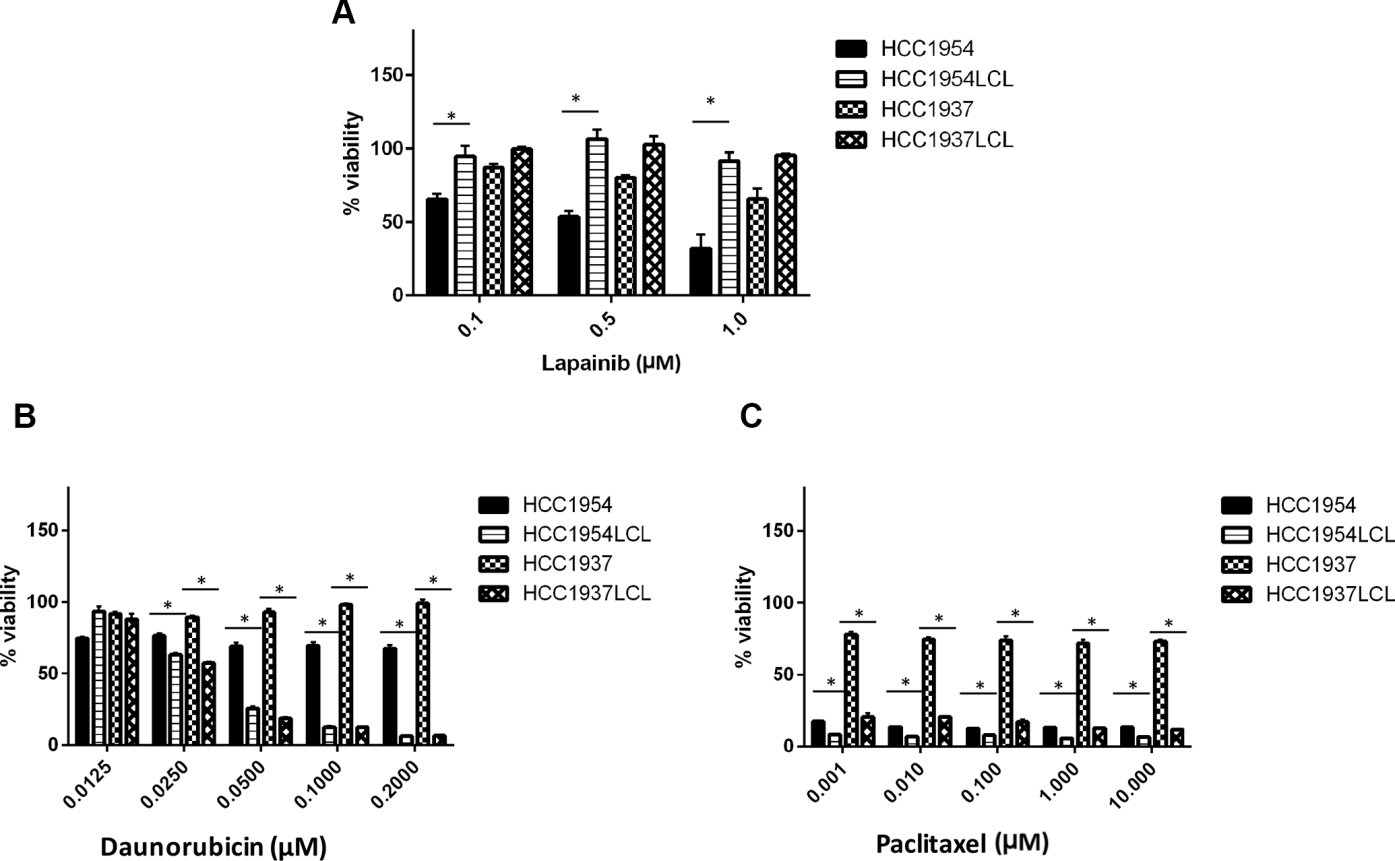
Effects of other anti-tumor drugs on 2 pairs of matching BC cell lines and LCLs HCC1954, HCC1937 and their matched LCLs were treated with (**A**) lapatinib (**B**) daunorubicin and (**C**) paclitaxel at their pharmacological concentrations. Percent cell viability was analyzed after 72 hours treatment with respective drugs. *p < 0.05.

In addition, we hypothesized that for drugs that are designed to target tumor-specific mutations and amplifications, LCLs will not be a good model to predict their tumor response. To test this we treated a pair of LCLs along with their matching breast cancer cell lines with lapatinib, an epidermal growth factor receptor (EGFR) and HER2 tyrosine kinase inhibitor. As expected, HCC1954, a HER2-amplified breast cancer cell line, was highly sensitive to lapatinib compared to its matched HCC1954 LCL line (*P* < 0.05, Figure 3A). Not surprisingly, in a second breast cancer cell line, HCC1937 (estrogen receptor (ER) and HER-2 negative), we observed very little sensitivity to lapatinib; and no sensitivity in the LCL derived from the same patient (Figure 3A). The minimal sensitivity of the cell line HCC1937 to lapatinib, an EGFR an HER2 inhibitor may be explained by its expression of EGFR [14, 15].

To further explore whether the observed response correlation between LCLs and cancer cell lines could be expanded to other cytotoxic drugs, we evaluated two other cytotoxic agents (daunorubicin and paclitaxel), and found that HCC1937 LCLs were more sensitive to daunorubicin and paclitaxel treatment than its matched HCC1937 breast cancer cell line (Figure 3B, 3C). HCC1954 LCL similarly showed more sensitivity to daunorubicin than its matched HCC1954 breast cancer cell line. Interestingly, under paclitaxel treatment, we observed similar high sensitivity in both HCC1954 breast cancer cell line and LCL from the same patient (HCC1954 LCL). This was consistent with the previous report that patients with a HER2-negative breast cancer benefitted from paclitaxel, regardless of estrogen-receptor status, but paclitaxel treatment did not benefit patients with HER2-negative cancers (like that of HCC1937) [16]. Overall, our data suggest that a germline model (like LCLs) may be used to predict tumor sensitivity to cytotoxic agents, while characterizing sensitivity to targeted therapy like lapatinib in LCLs is unlikely to be of use in predicting tumor sensitivity.

## DISCUSSION

The International HapMap LCLs have been extensively used to identify genetic predictors of chemotherapy toxicity and sensitivity including 5′-DFUR [9, 17, 18]. However, HapMap cells were derived from apparently healthy individuals. We therefore first sought to investigate whether we could establish LCLs from breast cancer patients. Our goal of establishing patient-derived LCLs was successful (> 85% success rate). We showed that LCLs can be established from patient PBMCs isolated freshly or from whole blood preserved on ice and isolated with 48 hours. This supports the feasibility on incorporating LCL establishment in clinical trials, even those across multiple sites. In addition, we demonstrated that our method to develop LCLs resulted in highly reproducible data with minimal intra-individual variability in response to 5′-DFUR treatment. This allowed observation of inter-individual variability in response to 5′-DFUR across different individuals. Given that laboratory-established LCLs can be cryopreserved (this will store donors' genetic and gene expression information at the time of collection), we created a research resource that can be used for future experiments evaluating the genetic contribution to drug sensitivity. Furthermore, the drugs that may be evaluated in this model in the future are not limited to capecitabine.

We next evaluated the role of patient-derived LCLs in predicting both short term and long term capecitabine treatment outcomes. Consistent with responses in previous studies, approximately 26 percent of patients in our study achieved CR+PR on single-agent capecitabine therapy [3]. We found no statistically significant correlation between capecitabine sensitivity derived from patient LCLs and their short term (10 to 12 weeks) response. However, there is a trend of positive correlation between them. This relationship is consistent with what was observed between *ex vivo* phenotype and long term response (represented by PFS). The lack of association may be due to the relatively small sample size of our correlative study, the short duration of treatment before response assessment, and/or decreased statistical power when evaluating categorical variable (ie, RECIST category) as compared to continuous variable (ie, PFS). Upon checking patients' medical record, most patients remained on capecitabine treatment even after the end of the trial, as well as those who received dose reductions.

Several clinical trials have shown that capecitabine significantly prolongs overall survival and PFS in the metastatic setting. Therefore, we also evaluated the correlation between the *ex vivo* 5′-DFUR sensitivity phenotype to patients' PFS. We found that patients with lower AUCs (higher *ex vivo* 5′-DFUR sensitivity) in LCLs had a longer PFS than patients whose LCLs had higher AUCs. Despite the confounder of the variable dose reductions that patients received due to toxicities, studies have also shown that lower dosage of capecitabine does not affect overall efficacy of therapy [19, 20]. Previous studies have shown that age and hepatic metastasis are independent prognostic factors of capecitabine response [21]. When we adjusted for these variables, patient-derived LCL sensitivity data remained an independent, significant predictor of patient outcomes.

This significant correlation between patients' *ex vivo* LCL and clinical outcome was further supported by an independent *in vitro* study of a collection of breast cancer cell lines with their matching LCLs. Our data seem to suggest that those shared germline elements between LCLs and tumor cells are important in cellular sensitivity to the cytotoxic agents.

Overall, we demonstrated that establishing LCLs from breast cancer patients is feasible and potentially beneficial. If further validated, our data supports the utility of patient-derived LCLs as a predictive model for capecitabine treatment efficacy in breast cancer patients. We showed a shared response to cytotoxic agents among LCLs and breast cancer cells derived from the same individuals. Further development and optimization of patient-derived LCLs or other germline, blood-based *ex vivo* models might provide powerful tools in precision medicine to tailor patients' therapies.

## MATERIALS AND METHODS

### Patients and clinical data collection

We conducted our study as part of a larger clinical trial (TBCRC 015, NCT00977119) in which women with metastatic breast cancer were enrolled in a clinical trial examining genetic determinants of capecitabine toxicity. The dose of capecitabine prescribed was standardized in all patients as 2000 mg/m^2^/day. For our correlative study, a subset of patients enrolled from six sites (University of Chicago, University of Alabama, Vanderbilt University, The University of Texas MD Anderson Cancer Center, University of Michigan and University of California - San Francisco) were included with the goal to evaluate the feasibility of creating *ex vivo* LCL models from breast cancer patients, and how these *ex vivo* drug sensitivity phenotypes related to patient capecitabine treatment response/toxicity phenotypes. The study was approved by the IRB of all participating institutions and informed consent was obtained from all study participants.

Both short term and long term clinical responses were collected. The short term response was measured at 10–12 weeks capecitabine treatment; while the long term response was assessed using PFS, which range from 6 weeks to 32 months. Clinical assessment and radiographic evaluation were performed on the majority of the patients at 12-week after initiation of capecitabine to assess their short term disease response. Response evaluation criteria in solid tumor (RECIST v1.1) criteria were applied and patient short term response was defined as complete response (CR), partial response (PR), stable disease (SD) or progressive disease (PD).

### Peripheral blood mononuclear cells isolation and LCL establishment

Ten milliliters of blood were drawn from each patient through venous puncture into BD Vacutainer^®^ venous blood collection tubes (Lavender top), and inverted 8–10 times. Blood samples were processed immediately (for all University of Chicago samples) or shipped on ice overnight to University of Chicago for processing. Peripheral blood mononuclear cells (PBMCs) were isolated using Accuspin™ System-Histopaque^®^-1077 tubes as instructed by the manufacturer (Sigma-Aldrich^®^, St. Louis, MO) with some modifications.

A previously-described Epstein-Barr virus (EBV) transformation protocol [22] was adapted to establish LCLs from breast cancer patients enrolled into our study. When the cells reached a total viability of 80% the flasks were sub-cultured and further expanded to a total viable cell count of 3 × 10^7^. Two independent LCL colonies were established from each patient's peripheral blood sample.

### Breast cancer cell lines and LCLs derived from the same breast cancer donors

A collection of breast cancer cell lines (*n* = 8) and their paired EBV transformed LCLs (*n* = 8) were established previously [13] and obtained from the American Tissue Culture Collection (ATCC^®^) (Manassas, VA). Of the breast cancer cell lines, 2 were from patients who had human epidermal growth factor receptor 2 (HER2+) cancers (HCC1954 and HCC2218), 1 was from a patient with an estrogen receptor positive (ER+) tumor (HCC1428) and 5 (HCC1937, HCC1143, HCC1187, HCC1599, HCC1395) were from patients who had triple negative breast cancers. Both the breast cancer cell lines and the matching LCLs were cultured according to ATCC protocol [13].

### *Ex vivo* phenotyping

Patient-derived LCLs from the TBCRC trial and the ATCC purchased cell lines (both breast cancer cell lines and their matching LCLs from the same donors) were phenotyped for 5′-DFUR sensitivity using Cell Titer Glo^®^ (Promega, Madison WI) [9]. Because LCLs lack the expression of cytidine deaminase, an enzyme critical for the conversion of capecitabine to its active form, 5′-DFUR (10 μM, 20 μM, 40 μM, 80 μM and 160 μM), a major metabolite of capecitabine was used to evaluate capecitabine sensitivity in cell growth inhibition assays. Cells with > 85% viability were plated in triplicate at 4000 cells per well in a 96-well plate (Corning, Corning NY). Cells were incubated with various concentrations of drug or vehicle control for 72 hours prior to the addition of Cell Titer Glo reagent in order to measure ATP levels in the culturing media. The area under curve (AUC) representing overall cellular sensitivity to the drug was calculated using the trapezoidal rule. For the matching breast cancer cell lines and LCLs, varying (pharmacologically achievable) concentrations of lapatinib (0.1–1 μM), daunorubicin (0.0125–0.2 μM) and paclitaxel (0.001–10 μM) were used to treat cells to confirm model validity. Each experiment was repeated at least 2 times in triplicate.

### Statistics

Linear regression was performed between *in vivo* RECIST defined patients' response at 12 weeks and response to 5′-DFUR in patient-derived LCLs (represented by the area under the curve (AUC)). Kaplan Meier analysis on patients progression-free survival (PFS) was used to evaluate difference between LCLs that are more sensitive (*n* = 17 low AUC) or resistant (*n* = 18 high AUC) to 5′-DFUR. Cox regression was used to adjust for multiple variables such as hepatic metastasis and age. Student‘s *T*-test was used to compare cellular response to 5′-DFUR between patients' LCLs and tumor cell lines. Pearson correlation was also used to evaluate correlation in 5′-DFUR sensitivity between patient-derived LCLs and their matched breast cancer cell lines.

## SUPPLEMENTARY MATERIALS FIGURES


