# Supplementary material for: Utility of patient-derived lymphoblastoid cell lines as an ex vivo capecitabine sensitivity prediction model for breast cancer patients
Source: Oncotarget. 2016 May 20;7(25):38359–66. doi: 10.18632/oncotarget.9521 (PMC5122395; doi:10.18632/oncotarget.9521)
Supplement: Supplementary file 1 [file oncotarget-07-38359-s001.pdf]

Utility of patient-derived lymphoblastoid cell lines as an *ex vivo* capecitabine sensitivity prediction model for breast cancer patients

Supplementary Materials

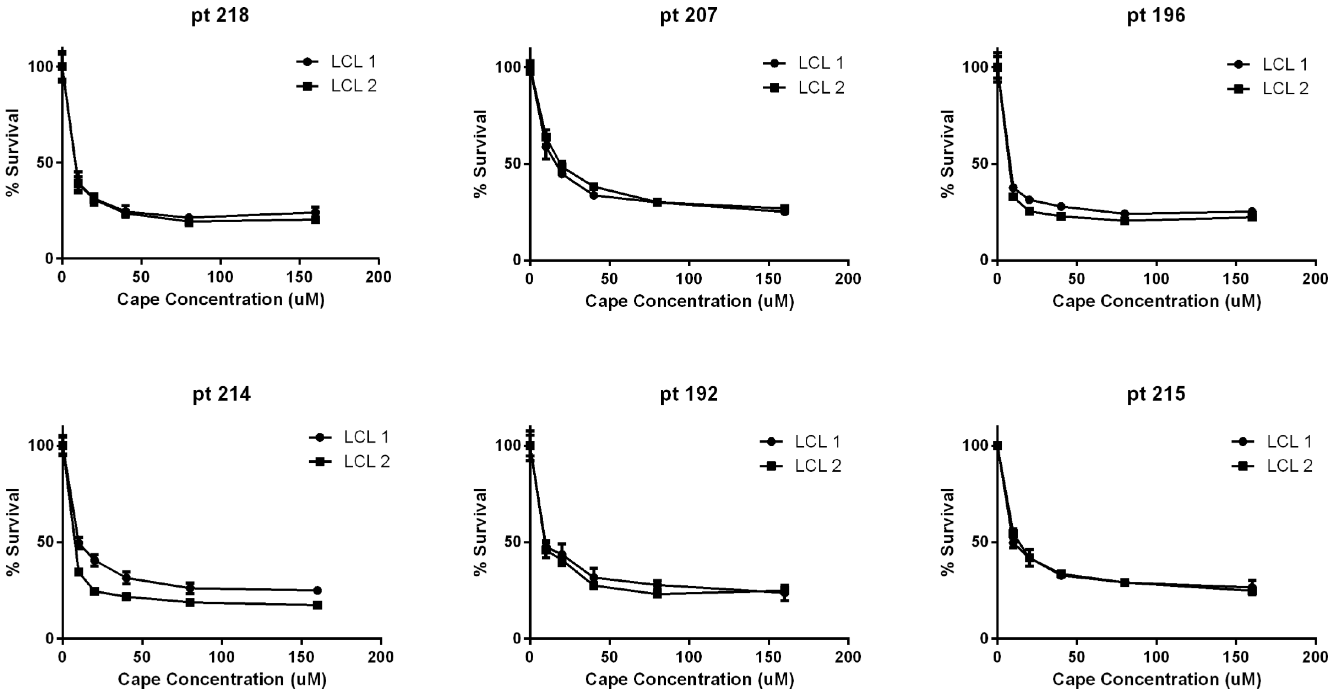

Supplementary Figure S1: Minimal Intra-individual variability in the transformation process from patient PBMCs to LCLs.

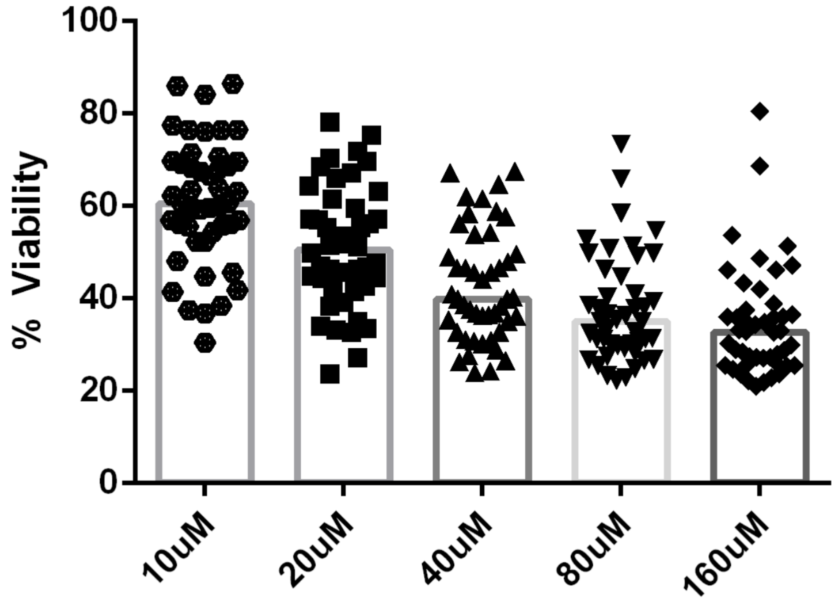

Supplementary Figure S2: patient-derived LCLs sensitivity to 5′DFUR.

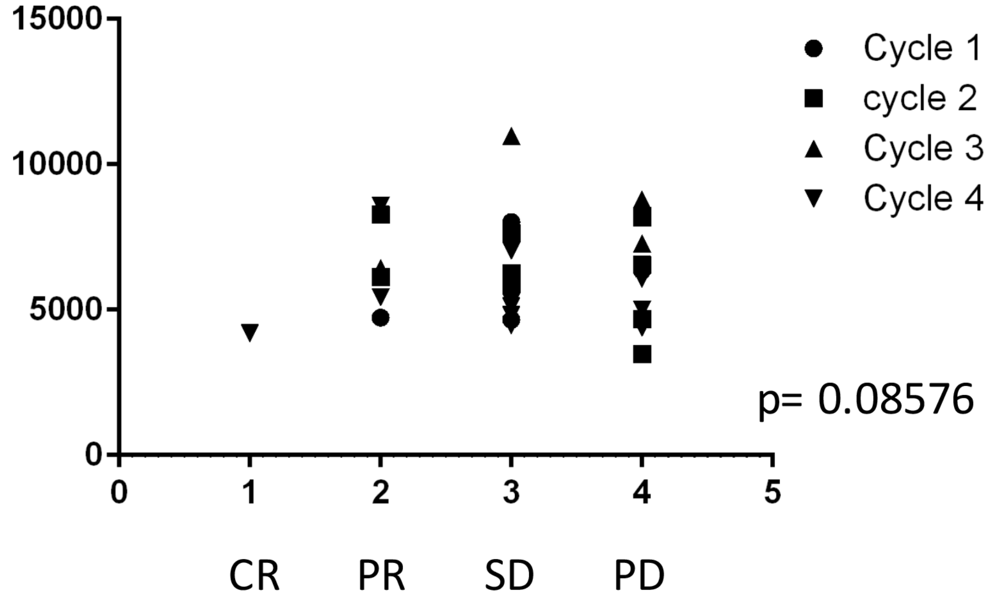

Supplementary Figure S3: Patient-derived LCLs does not correlate with patient short term response.

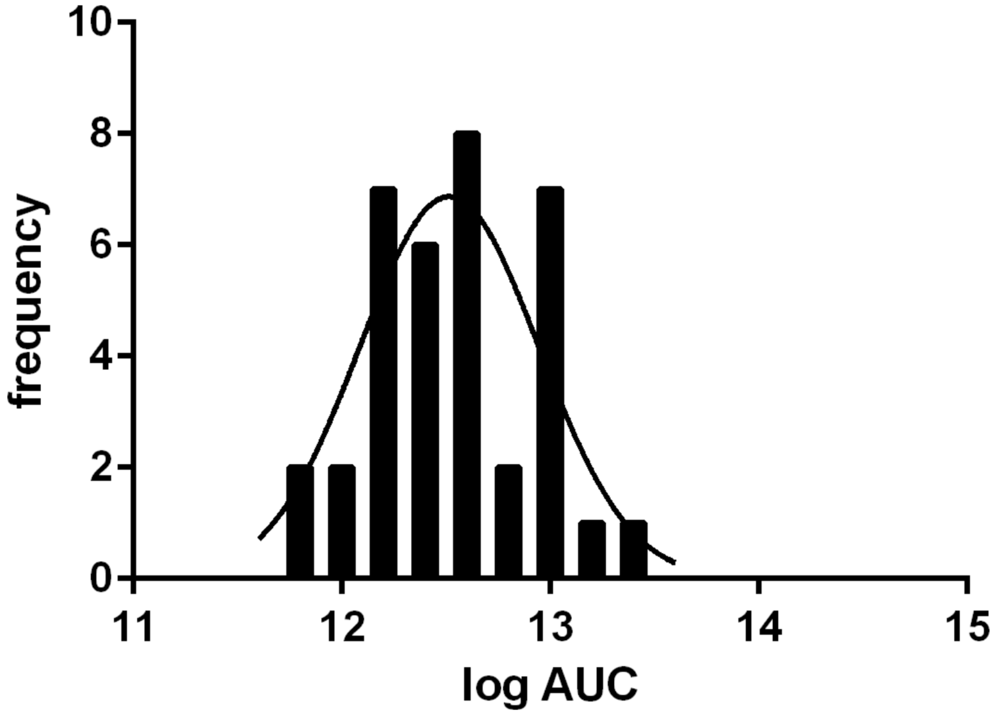

Supplementary Figure S4: Frequency distribution of patient-derived LCL AUC of 5′-DFUR
